# Human Polyomaviruses in Children Undergoing Transplantation, United States, 2008–2010

**DOI:** 10.3201/eid1810.120359

**Published:** 2012-10

**Authors:** Erica A. Siebrasse, Irma Bauer, Lori R. Holtz, Binh-minh Le, Sherry Lassa-Claxton, Charles Canter, Paul Hmiel, Shalini Shenoy, Stuart Sweet, Yumirle Turmelle, Ross Shepherd, David Wang

**Affiliations:** Washington University School of Medicine, St. Louis, Missouri, USA

**Keywords:** transplant, polyomavirus, immunosuppression, Polyomaviridae, viruses, children, pediatric

## Abstract

Immunocompromised patients are at risk for disease caused by infection by some polyomaviruses. To define the prevalence of polyomaviruses in children undergoing transplantation, we collected samples from a longitudinal cohort and tested for the 9 known human polyomaviruses. All were detected; several were present in previously unreported specimen types.

BK and JC polyomaviruses (BKPyV, JCPyV) cause disease in immunocompromised persons. Both are double-stranded DNA viruses in the family *Polyomaviridae*. Seven additional human polyomaviruses were discovered during 2007–2011: KI polyomavirus (KIPyV) (*1*), WU polyomavirus (WUPyV) (*2*), Merkel cell polyomavirus (MCPyV) (*3*), human polyomavirus 6 (HPyV6) (*4*), human polyomavirus 7 (HPyV7) (*4*), trichodysplasia spinulosa-associated polyomavirus (TSPyV) (*5*), and human polyomavirus 9 (HPyV9) (*6*).

The 7 novel polyomaviruses have been detected in various specimen types; detection has been extensively reviewed for KIPyV, WUPyV, and MCPyV (*7*). Polyomaviruses HPyV6, HPyV7, TSPyV, and HPyV9 have been detected in skin (*4*,*5*,*8*); TSPyV and HPyV9 have also been detected in urine, and HPyV9 was detected in serum (*6*). However, only 2 of these recently identified viruses have been specifically implicated in human diseases; MCPyV is associated with Merkel cell carcinoma (*3*), and TSPyV has been linked to trichodysplasia spinulosa (*5*). Immunosuppression is a likely cofactor in both diseases. The potential pathogenicity of the other 5 novel polyomaviruses is unknown. As a first step toward exploring their disease potential, we sought to define their prevalence in immunocompromised transplant recipients. To this end, we established a longitudinal cohort of children undergoing transplantation at St. Louis Children’s Hospital, St. Louis, Missouri, USA.

## The Study

We recruited 32 patients who were scheduled to receive transplants (2 lung, 11 liver, 5 heart, 2 kidney, 1 liver/lung, and 11 bone marrow transplants) during October 2008–April 2010. The Human Research Protection Office of Washington University in St. Louis approved this study. The mean age of enrolled patients was 5.8 years, and the median age was 3.1 years. Thirty patients received transplants and were studied for 1 year after transplantation. We collected 716 clinical specimens (160 nasopharyngeal swab, 169 urine, 122 fecal, 265 plasma) during 265 patient visits. We collected 298 specimens from patients during symptomatic episodes, which were defined as having >1 of the following: fever, respiratory symptoms, or gastrointestinal symptoms. We collected clinical data using a questionnaire and the medical records.

Fecal material was diluted 1:6 in phosphate-buffered saline and filtered through 0.45-μm membranes. For all specimens, we extracted total nucleic acids using an Ampliprep Cobas extractor (Roche, Branchburg, NJ, USA). We used published real-time PCRs for WUPyV (*9*), KIPyV (*9*), TSPyV (*5*), MCPyV (*10*), BKPyV (*11*), and JCPyV (*12*) (Table 1). We developed assays for HPyV6, HPyV7, and HPyV9 using Primer Express software (Applied Biosystems, Carlsbad, CA, USA) (Table 1). To assess the performance of each assay, we used serial dilutions (5 to 5 × 10^6^ copies/reaction) of a plasmid containing the target sequence. All 3 assays demonstrated a sensitivity of ≈5 copies/reaction and yielded linear curves with R^2^ values >0.99.

**Table 1:** Real-time PCR assays to detect human polyomaviruses in children undergoing transplants, United States, 2008–2010*

Virus	Target	Primers, 5′ → 3′	Probe, 5′ → 3′
WUPyV	NCCR	WU-C-4824-F: GGCACGGCGCCAACT	WU-C-4861-TM: FAM-TGCCATACCAACACAGCTGCTGAGC-TAMRA-3′
		WU-C-4898-R: CCTGTTGTAGGCCTTACTTACCTGTA	
KIPyV	LTAg	KI-B-4603-F: GAATGCATTGGCATTCGTGA	KI-B-4632-TM: FAM-TGTAGCCATGAATGCATACATCCCACTGC-TAMRA
		KI-B-4668-R: GCTGCAATAAGTTTAGATTAGTTGGTGC	
TSPyV	LTAg	LTF: TGTGTTTGGAAACCAGAATCATTTG	LTP: FAM-TTCTTCTTCCTCCTCATCCTCCACCTCAAT-TAMRA
		LTR: TGCTACCTTGCTATTAAATGTGGAG	
MCPyV	LTAg	LT.1F: CCACAGCCAGAGCTCTTCCT	LT probe: FAM-TCCTTCTCAGCGTCCCAGGCTTCA-TAMRA
		LT.1R: TGGTGGTCTCCTCTCTGCTACTG	
HPyV6	VP1	ES011F: GCCTGGAAGGGCCTAGTAAAG	ES024: FAM-ACCAACCATCTGTTGCAGGCATTAAAGCTA-TAMRA
		ES012R: ATTGGCAGCTGTAACTTGTTTTCTG	
HPyV7	VP1	ES017F: GGTCCAGGCAATACTGATGTAGCTA	ES025: FAM-CCTGCAAGCCCTCAGAAAGCAAGTAAATG-TAMRA
		ES018R: TCTGCAACCCAGAGCTCTACTG	
HPyV9	LTAg	ES026F: GAAGACCCTGATCCTGAGGAAGA	ES031: FAM-TGGATCATCCCAGAGTTCATTTACCTGCA-TAMRA
		ES027R: CTCTGGAGTATTAGGTTCAGGCTTCT	
BKPyV	LTAg	BK-Deg-F: AGCAGGCAAGRGTTCTATTACTAAAT	Bk-Deg-P: FAM-AAGACCCTAAAGACTTTCCYTCTGATCTACACCAGTTT-TAMRA
		BK-Deg-R: GARGCAACAGCAGATTCYCAACA	
JCPyV	VP2/3	JL1 (F): AAGGGAGGGAACCTATATTTCTTTTG	JL1 (P): FAM-CTCATACACCCAAAGTATAGATGATGCAGACAGCA-TAMRA
		JL1 (R): TCTAGCCTTTGGGTAACTTCTTGAA	

*WUPyV, WU polyomavirus; NCCR, non-coding control region; KIPyV, KI polyomavirus; LTAg, large T antigen; TSPyV, trichodysplasia spinulosa polyomavirus; MCPyV, Merkel cell polyomavirus; HPyV, human polyomavirus; VP, virion protein; BKPyV, BK polyomavirus; JCPyV, JC polyomavirus.

Each of the 25-μl quantitative PCRs included 5 μL of extracted sample, 12.5 pmol of each primer, and 4 pmol of probe. The MCPyV primers and probe were used as described (*10*). We tested samples in a 96-well plate format, with 8 water negative controls and 1 positive control/plate. Reactions were cycled as recommended using either an ABI 7500 real-time thermocycler (Applied Biosystems) or a CFX96 real-time thermocycler (BioRad, Hercules, CA, USA). The threshold of all plates was set at a standard value, and samples were counted as positive if their cycle threshold was <37.00.

All 716 specimens were tested for each virus (Table 2). The most frequently detected virus was BKPyV, which was found primarily in urine as expected. JCPyV was detected in 1 plasma sample. HPyV6, HPyV7, MCPyV, and TSPyV were detected in specimen types not previously reported. HPyV6 and TSPyV were detected in fecal samples and nasopharyngeal swab samples, and HPyV7 was detected in a nasopharyngeal swab and urine. One fecal sample was positive for MCPyV. Because HPyV6, HPyV7, and MCPyV have been previously detected in skin, we cannot rule out the possibility that their presence in specimens could have been caused by shedding from skin.

**Table 2:** Polyomaviruses detected among specimens from children undergoing transplants, United States, 2008–2010*

Virus	Specimen type	Transplant	C_t_	Patient ID	Date of collection, time elapsed from transplant
HPyV6	Feces	BMT	32.19	3011	2012 Jun 06, 1 mo after transplant
HPyV6	NP	Heart	36.13	4005	2010 Nov 25, 7 mo after transplant
HPyV6	Feces	Lung	36.95	5001	2010 Aug 17, 1 mo after transplant
HPyV7	NP	Liver	34.57	1002	2009 Jun 16, 7 mo after transplant
HPyV7	Urine	Liver	36.54	1002	2009 Jul 15, 8 mo after transplant
HPyV9	Urine	Liver	36.72	1009	2010 Feb 09, 1 wk after transplant
KIPyV	NP	BMT	16.28	3001	2009 Jul 07, 3 mo after transplant
KIPyV	NP	BMT	36.07	3001	2009 May 19, 1 mo after transplant
KIPyV	NP	BMT	33.37	3008	2009 Nov 12, before transplant
KIPyV	NP	BMT	31.04	3009	2010 Jul 30, 6 mo after transplant
MCPyV	NP	BMT	36.29	3011	2010 Apr 15, before transplant
MCPyV	Feces	BMT	34.56	3011	2010 Jul 02, 2 mo after transplant
TSPyV	NP	Heart	32.98	4001	2009 May 29, 1 wk after transplant
TSPyV	NP	Heart	30.74	4001	2009 Jun 18, 1 mo after transplant
TSPyV	Feces	Heart	33.89	4001	2009 May 29, 1 wk after transplant
WUPyV	NP	BMT	36.62	3005	2009 Jul 15, before transplant
WUPyV	NP	BMT	28.81	3007	2009 Nov 06, 2 mo after transplant
JCPyV	Plasma	BMT	36.12	3011	2010 Aug 24, 3 mo after transplant
BKPyV	Urine	BMT	15.83	3010	2010 Apr 15, 1 mo after transplant
BKPyV	Urine	Kidney	36.67	2022	2010 Jul 01, 10 mo after transplant
BKPyV	Urine	BMT	30.80	3011	2010 Aug 24, 3 mo after transplant
BKPyV	Urine	Heart	25.84	4001	2009 Aug 14, 2 mo after transplant
BKPyV	Urine	Heart	35.89	4003	2009 Dec 23, 2 mo after transplant
BKPyV	Urine	Heart	24.37	4001	2009 Sep 23, 4 mo after transplant
BKPyV	Urine	Liver	28.56	1010	2009 Nov 23, 1 wk after transplant
BKPyV	Urine	Lung	33.13	5002	2011 May 03, 1 year after transplant
BKPyV	Urine	Lung	25.25	5002	2011 Feb 08, 10 mo after transplant
BKPyV	Urine	Kidney	9.97	2002	2010 Mar 04, 6 mo after transplant
BKPyV	Urine	BMT	30.10	3009	2010 Mar 05, 2 mo after transplant
BKPyV	Urine	Liver	22.89	1001	2009 Jan 07, 3 mo after transplant
BKPyV	Urine	Kidney	34.41	2002	2010 May 13, 8 mo after transplant
BKPyV	NP	Kidney	35.93	2002	2010 Mar 04, 6 mo after transplant
BKPyV	Feces	Kidney	33.15	2002	2010 Mar 04, 6 mo after transplant
BKPyV	Feces	Liver	33.33	1001	2008 Dec18, 2 mo after transplant
BKPyV	Feces	Liver	34.84	1001	2009 Jan 07, 3 mo after transplant

*C_t_, cycle threshold; ID, identification; HPyV, human polyomavirus; BMT, bone marrow transplant; NP, nasopharyngeal; KIPyV, KI polyomavirus; MCPyV, Merkel cell polyomavirus; TSPyV, trichodysplasia spinulosa polyomavirus; WUPyV, WU polyomavirus; JCPyV, JC polyomavirus; BKPyV, BK polyomavirus

We collected 2 serial nasopharyngeal samples that were positive for KIPyV from patient 3001 (Table 2), a 1-year-old child who had received a bone marrow transplant as treatment for Fanconi anemia. The first sample, a nasopharyngeal swab obtained 1 month after transplant, had low levels of KIPyV. To determine the viral load of the second nasopharyngeal swab specimen collected 2 months later, we reanalyzed the sample in triplicate; on the basis of extrapolation of the standard curve run in parallel, we estimated the viral load to be 1.3 × 10^9^ genome copies/mL of nasopharyngeal swab transport media. This patient’s course was complicated by graft-versus-host disease of the gut and skin, renal failure requiring dialysis, and recurrent pulmonary hemorrhage. The patient was critically ill and had experienced multiorgan failure at the time of the second sampling. Other microbiological test results were negative at that time, including PCR for Epstein-Barr virus, cytomegalovirus, human herpesvirus-6, and adenovirus in the blood; aspergillus antigen detection in blood; and bacterial cultures of blood, tracheal aspirate, urine, and peritoneal fluid. The fecal specimen collected at this time was negative for KIPyV; plasma and urine were not available for this study. The patient died of acute respiratory failure and extensive pulmonary hemorrhage 24 days after collection of this specimen. Despite the frequent detection of KIPyV in respiratory specimens, no studies have definitively linked infection with respiratory disease. Titers of KIPyV were high in the nasopharyngeal swab sample from this patient 3 weeks before respiratory failure. Although this observation does not necessarily implicate KIPyV infection as a contributing factor in the death of the patient, it suggests a poorly controlled KIPyV infection in the respiratory tract.

Three specimens collected from patient 4001, a 13-year-old heart transplant recipient, were positive for TSPyV (Figure), but the patient did not have trichodysplasia spinulosa. At 1 week after transplant, the nasopharyngeal swab and fecal samples were positive for TSPyV. At 1 month after transplant, the nasopharyngeal swab sample was again positive for TSPyV, with a viral load of ≈2.3 × 10^4^ genome copies/mL of transport media. There is currently only 1 TSPyV sequence in GenBank (accession no. GU989205). We used 4 primer pairs to amplify the complete genome of TSPyV from the nasopharyngeal swab taken 1 month after transplant. PCR products were cloned, and the complete genome was sequenced to 3× coverage (GenBank accession no. JQ723730) and compared with the other TSPyV sequence. There were 5 nt substitutions: 3 in noncoding regions and 2 synonymous mutations.

**Figure:**
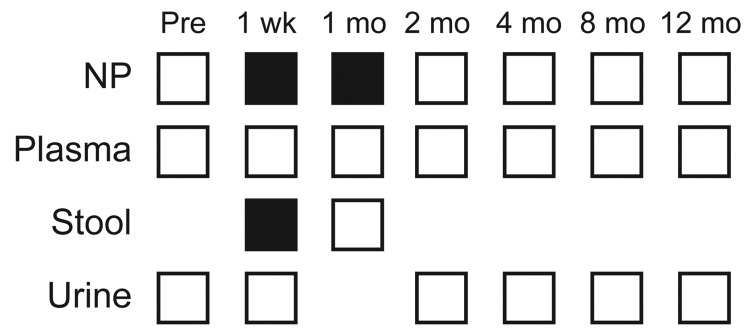
Samples tested for TSV (trichodysplasia spinulosa polyomavirus) during May–June 2009 from patient 4001, a 13-year-old heart transplant recipient at St. Louis Children’s Hospital, St. Louis, Missouri, USA. Samples tested at each time point are indicated by white squares. Black squares represent positive samples. NP, nasopharyngeal.

Although serologic studies have demonstrated that ≈70% of adults in Europe have been infected by TSPyV (*13*), its mode of transmission is unknown. The detection of TSPyV in nasopharyngeal swab and fecal samples raises the possibility that it may be transmitted by a respiratory or fecal–oral route. Furthermore, in the current study, 2 sequential nasopharyngeal swab samples taken 20 days apart were positive for TSPyV, suggesting it may persist for extended periods in the respiratory tract, at least in immunosuppressed persons.

## Conclusions

Our goals were to establish a longitudinal repository of different specimens types from transplant recipients and to define the prevalence of polyomaviruses in these patients. We detected all 9 polyomaviruses in at least 1 specimen. Although the prevalence of each virus was generally low, TSPyV, HPyV6, HPyV7, and MCPyV were detected in specimen types not previously reported. These observations expand understanding of the recently identified polyomaviruses and the tissue and organ systems they may infect and suggest possible modes of transmission. Further studies to define their possible roles in human diseases are needed.
